# Development of a wireless ultrasonic brain stimulation system for concurrent bilateral neuromodulation in freely moving rodents

**DOI:** 10.3389/fnins.2022.1011699

**Published:** 2022-09-23

**Authors:** Evgenii Kim, Jeungeun Kum, Seung Hyun Lee, Hyungmin Kim

**Affiliations:** ^1^Biomedical Research Division, Bionics Research Center, Korea Institute of Science and Technology, Seoul, South Korea; ^2^Division of Bio-Medical Science and Technology, KIST School, Korea University of Science and Technology, Seoul, South Korea

**Keywords:** ultrasound, neuromodulation, low-intensity, bilateral, interhemispheric connection

## Abstract

Bilateral brain stimulation is an important modality used to investigate brain circuits and treat neurological conditions. Recently, low-intensity pulsed ultrasound (LIPUS) received significant attention as a novel non-invasive neurostimulation technique with high spatial specificity. Despite the growing interest, the typical ultrasound brain stimulation study, especially for small animals, is limited to a single target of sonication. The constraint is associated with the complexity and the cost of the hardware system required to achieve multi-regional sonication. This work presented the development of a low-cost LIPUS system with a pair of single-element ultrasound transducers to address the above problem. The system was built with a multicore processor with an RF amplifier circuit. In addition, LIPUS device was incorporated with a wireless module (bluetooth low energy) and powered by a single 3.7 V battery. As a result, we achieved an ultrasound transmission with a central frequency of 380 kHz and a peak-to-peak pressure of 480 kPa from each ultrasound transducer. The developed system was further applied to anesthetized rats to investigate the difference between uni- and bilateral stimulation. A significant difference in cortical power density extracted from electroencephalogram signals was observed between uni- and bilateral LIPUS stimulation. The developed device provides an affordable solution to investigate the effects of LIPUS on functional interhemispheric connection.

## Introduction

A large body of research suggests that bilateral brain stimulation may provide therapeutical benefits for various neurological disorders (Kumar et al., 1999). In clinical settings, bilateral deep brain stimulation (DBS) has shown improvements in motor deficits and quality of life in patients with advanced Parkinson’s conditions (Williams et al., 2010; Odekerken et al., 2013). Invasive electrical stimulation of two thalami is sought when unilateral stimulation cannot provide satisfactory therapeutic effects for essential tremors (Ondo et al., 2001). Additionally, pharmacologically resistant major depressive disorder is within the scope of bilateral DBS utility (Mayberg et al., 2005; Taghva et al., 2013). However, due to the invasive nature of DBS, the risk of postoperative complications is increased with an additional implanted electrode (Voges et al., 2007). Non-invasive bilateral activation of motor cortices by transcranial magnetic stimulation (TMS) showed a decrease in the Yale Global Tic Severity Scale in children with Tourette syndrome (Kahl et al., 2021), as well as an enhancement of motor function after a stroke (Takeuchi et al., 2009; Park et al., 2017). Another non-invasive technique is transcranial direct current stimulation (tDCS) predominantly affects both hemispheres and provides an additional therapeutic option for addiction (Batista et al., 2015; Kim and Kang, 2021) and motor-related disorders (Mordillo-Mateos et al., 2012; Di Lazzaro et al., 2014). Despite the broad application of TMS and tDCS, they suffer from poor spatial precision and shallow penetration depths (Wagner et al., 2007). Therefore, alternative neurostimulation techniques that can overcome these limitations are in demand.

Recent advances in focused ultrasound (FUS) have found broad applications in neurotherapeutic scenarios (Rezayat and Toostani, 2016; Meng et al., 2021). FUS offers relatively high precision and has been successfully applied to deep brain regions in a non-ionizing, non-invasive fashion (Legon et al., 2018; Shin et al., 2018). High-intensity transcranial ultrasound has been used in clinical studies to provide localized thermal ablation of deep brain tissues to treat tumors (Zhou, 2011), essential tremors (Schreglmann et al., 2017), and Parkinson’s disease (Dobrakowski et al., 2014). Meanwhile, low-intensity pulsed ultrasound (LIPUS) showed a non-thermal, non-destructive capability to stimulate brain circuits (Rezayat and Toostani, 2016; Huang et al., 2019). Furthermore, varying the acoustic pulsing scheme resulted in the suppression or excitation of neural networks (Yoo et al., 2011; Kim et al., 2015; Yoon et al., 2019). *In vivo* functional magnetic resonance imaging (fMRI) (Verhagen et al., 2019) and *ex vivo* whole-cell patch-clamp recording (Clennell et al., 2021) showed that acoustic neuromodulation offers long-lasting effects, thereby opening an unprecedented potential for various neurological disease interventions. LIPUS has already been used to facilitate post-stroke recovery (Baek et al., 2018), to suppress seizure activities (Chen et al., 2020), and to treat major depression (Tsai, 2015). In clinical application, thalamic ultrasound stimulation was employed to treat consciousness disorders (Monti et al., 2016).

A typical LIPUS system consists of a single-element transducer that requires at least one off-the-shelf function generator and a linear RF amplifier (Tufail et al., 2011). Implementing a second transducer for bilateral stimulation would increase the system complexity and cost by about twofold. Moreover, excessive tethering of the transducers limits the motion of an animal subject on which the device is mounted, causing undesired psychological stress (Blanchard et al., 2001). A basic solution was suggested in a behavioral study involving non-human primates, in which sequential sonication was used on the right and left basal forebrain to alternate decisions (Khalighinejad et al., 2020). However, time delays prevalent in paired stimulation substantially impacted the stimulation outcome (Oliveri et al., 2000; Torii et al., 2019). The phased-array configuration has the potential for concurrent dual-target stimulation, but this approach is still limited by immobilized animals with a fixed, bulky acoustic probe above the head (Li et al., 2018), thus inducing potential physiological and behavioral changes which may interfere with the stimulation (Kim et al., 2017).

The above limitations motivated us to develop a wireless, low-cost system for concurrent LIPUS-mediated bilateral stimulation on rats. In the previous work, we developed a portable transcranial ultrasound system for remote brain stimulation of freely behaving animals (Kim et al., 2020). However, the system was designed to drive only a single ultrasound transducer, thus offering single-target stimulation. Herein, the bilateral brain stimulation system was constructed with a multicore processor providing separate control of two in-house built transducers. The efficacy of the developed method was examined on Sprague Dawley rats through electroencephalogram (EEG). In addition, the power density change of the EEG signal was analyzed to evaluate a functional interhemispheric communication induced by bilateral LIPUS stimulation.

## Materials and methods

### Ultrasound system

The ultrasound transducers were designed based on PZT-4 ceramics with a resonance frequency of 380 kHz and a diameter of 7 mm (SMD07T02R412WL, Steiner & Martins Inc., Davenport, FL, USA). PZTs were encapsulated into an individual 3D printed (Verowhite, Stratasys, Eden Prairie, MI, USA) transducer housing (8 mm in diameter, 6 mm in height). Then, a 5.5 mm thick thermoplastic adhesive (3764, 3M, Saint Paul, MN, USA) was filled on one side as an acoustic backing layer to absorb back-reflected sound waves.

**FIGURE 1:**
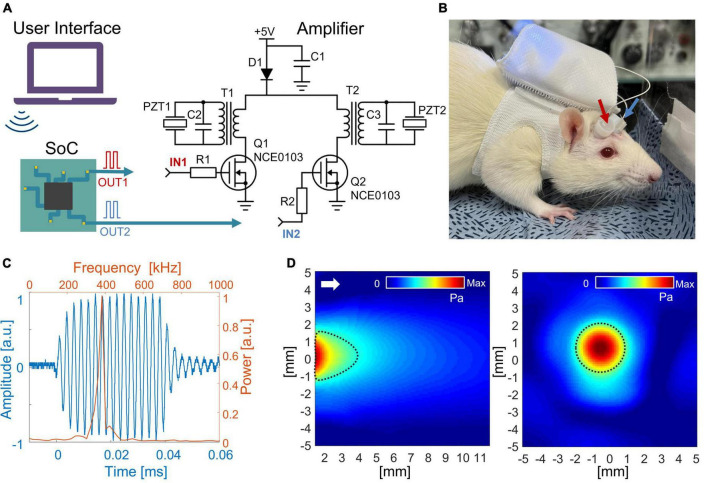
**(A)** Schematic diagram of the wireless LIPUS system. A system on a chip (SoC) generates PWM signals that are further converted to 380 kHz sine waves to feed ultrasound transducers (PZTs). R1 = R2 = 100 Ω; C1 = C2 = C3 = 100 uF; D1 – SS14. **(B)** The entire system fits inside a backpack that was worn by an awake rat. The arrows indicate ultrasound transducers. **(C)** An acoustic waveform with a central frequency of 380 kHz was generated from the LIPUS system. The time and frequency domains are shown as blue and orange color, respectively. **(D)** The acoustic beam profile of the in-house built transducer was measured in a free water condition. The longitudinal pressure map is on the left, and the transverse profile is on the right (measured 2 mm away from the transducer surface). The full width at half-maximum (FWHM) of pressure is indicated with dotted black lines. The white arrow shows the direction of sonication.

The driving system which controls transducers was developed based on two main compartments: a system on chip (SoC, nRF52840, NORDIC Semiconductor, Norway) and a pair of RF amplifier circuits. Firstly, a pulse width modulated (PWM) signal with a frequency of 380 kHz and 50% duty cycle (DC) was generated by a multicore processor inside the SoC. Then, the generated PWM signal was amplified and lowpass filtered by the amplifying circuit (Figure 1), which mainly consisted of a combination of a metal-oxide-semiconductor field-effect transistor (MOSFET; NCE0103, NcePower, China) and a transformer with 1:6 ratio between the primary and secondary winding. MOSFET with a transformer offered an initial voltage boost of PWM due to additional voltage originating across the primary coil of the transformer. Then, the signal was amplified a second time due to the difference in ratio between the primary and secondary winding. The voltage across PZT was measured using a digital oscilloscope (DSOX1204G, Keysight Technology, USA). Each core (total of four cores) produces the independent PWM waveform, offering the capability of each transducer to set different sonication schemes [i.e., pulse repetition frequency (PRF), pulse duration, and sonication onset]. The acoustic beam profile and waveform from each transducer were measured in free water space by needle-type hydrophone (HNR-0500, 0.5 mm probe, ONDA Corp., USA). The acoustic attenuation caused by the rat skull was measured by placing the freshly extracted rat skull between an ultrasound transducer and a hydrophone.

Bluetooth low energy (BLE) module was embedded into the SoC, offering a wireless control of stimulation protocol [i.e., stimulation onset, sonication duration (SD), PRF, and DC of stimulation]. The entire system was powered by a single 3.7 V lithium-polymer battery with 900 mAh, with a DC–DC power converter (IP5306) to boost 3.7–5 V. The entire system was compiled on a circuit board with dimensions 40 mm × 40 mm × 10 mm. The circuit with a total weight of 33 g (15 g excluding battery) was placed inside a rat’s backpack, offering the possibility for the animal to move freely (Figure 1B). For the awake rat, the transducers were transiently fixed over the scalp using topical skin adhesive (Dermabond, Johnson & Johnson, USA).

### Animal preparation

All animals were cared for in accordance with the guidelines for the Care and Use of Laboratory Animals. The animals were housed in a temperature-controlled room with alternate light/dark conditions (a 12 h light/dark cycle, light on 07:00–19:00) and *ad libitum* access to water and food. All surgical procedures were carefully reviewed and approved by the Institutional Animal Care and Use Committee (IACUC) of the Korea Institute of Science and Technology. Nine Sprague-Dawley rats (7–10 weeks old male, 220–280 g) were used for this study.

### Surgical procedures and electrophysiological assessment

Animals were anesthetized by an intramuscular injection of a ketamine/xylazine mixture (80 mg/kg ketamine, 10 mg/kg xylazine). Sufficient depth of anesthesia was ensured prior to the surgical procedure by toe pinch assessments. The additional dose of anesthetic agent (one-third of the original dose) was delivered as needed to complete the surgical and LIPUS procedures. The scalp fur was removed, and a midline incision was made to expose the skull. Six custom-made screw-type electrodes were fabricated with insulated copper wires (UL-AWG36, SME, South Korea), and anchor screws were fixed on the skull to record EEG signals. Two electrodes were placed above a motor cortex [Anteroposterior (AP): −3 mm, Mediolateral (ML): ±1 mm]; the other two were located above the somatosensory area (AP: −3 mm, ML: ± 4 mm); ground and reference electrodes were fixed above the cerebellum. EEG activity was measured from the rat cortex with an 8 kHz sampling frequency and finite impulse response bandpass filtering from 0.1 to 200 Hz (Digital Lynx SX, Neuralynx, Inc., USA). EEG was acquired with a wired connection for precise recording of neural signals, while control of LIFUS was performed wirelessly through BLE communication. Two transducers were placed above the right and left primary motor cortex (AP: +1.5 mm, ML: ±4 mm) and connected to the LIPUS system. The ultrasound gel was applied between the transducer and the skull to minimize acoustic impedance mismatching. A custom code in MATLAB (R2020a, MathWorks, Inc., USA) was used to control and synchronize LIPUS stimulations with EEG recording *via* Bluetooth protocol.

### Ultrasound stimulation protocols

The right primary motor (M1) cortex was selected to evaluate the effect of unilateral LIPUS stimulation with one of two sonication schemes: 1 kHz PRF and 50% DC (stimulation condition #1), 100 Hz PRF and 5% DC (stimulation condition #4). During the unilateral stimulation, the transducer on the left hemisphere did not generate any ultrasound wave.

**FIGURE 2:**
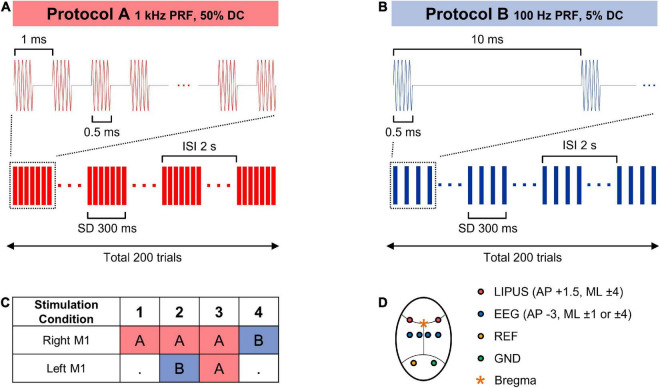
**(A,B)** Sonication parameters for the LIPUS stimulation. **(C)** Combinations of sonication parameters for unilateral (1, 4) and bilateral (2, 3) primary motor cortex stimulation. **(D)** A schematic diagram of LIPUS stimulation and EEG recording cite.

Two bilateral sonication protocols were integrated into the experiment procedure to evaluate the modulatory effect produced by simultaneous, contralateral LIPUS stimulation. Stimulation condition #2 combines excitatory stimulation (1 kHz PRF and 50% DC) on the right hemisphere with concurrent suppressive stimulation (100 Hz PRF and 5% DC) on the left motor cortex. During stimulation condition #3, both the right and left hemispheres underwent simultaneous excitatory stimulation (1 kHz PRF and 50% DC). All stimulation conditions were performed with a central frequency of 380 kHz, SD of 300 ms, inter-stimulus interval (ISI) of 2 s, and spatial-peak pulse-average intensity (I_*SPPA*_) of 2.3 W/cm^2^, as shown in Figure 2. Each sonication condition consists of 200 stimulation trials.

### Electrophysiological analysis

Electroencephalogram signal was analyzed based on the power density of brain wave oscillation. A notch filter with a cutoff frequency of 60 Hz was applied to remove line noise from the EEG signal. The time-locked signal of the EEG oscillation synchronized with the stimulation interval was collected from each stimulation trial. Trials with substantial noise were removed by a thresholding method. For each sonication condition, a total of 1,700 trials were randomly collected to match the same numbers for statistical analysis. Power spectral density (PSD) analysis was performed by the short-time Fourier transform (STFT) on each trial by sliding the 500 ms time window for every 16.7 s. The averaged across baseline (from −500 to −200 ms) and stimulation period (from 0 to 300 ms) value of delta (1–4 Hz), theta (4–8 Hz), alpha (8–13 Hz), low beta (13–20 Hz), high beta (20–30 Hz), and gamma (30–45 Hz) oscillation was collected from each trial. PSD was grand averaged within each sonication condition to evaluate the effect of the LIPUS stimulation on the neural activity change. A two-tailed, paired *t*-test was performed to compare baseline and LIPUS periods in terms of the power of brainwaves. PSD associated with LIPUS period (from 0 to 300 ms) from each trial was normalized with respect to baseline values and averaged within each sonication condition to compare the effect of LIPUS from each sonication condition. Kruskal–Wallis, non-parametric one-way ANOVA followed by the Tukey–Kramer *post hoc* analysis was used to compare the PSD change induced by stimulation conditions.

## Results

### Bilateral low-intensity pulsed ultrasound system

The voltage across PZT showed the capability of the developed system to convert 3.7 V PWM from SoC to a half-rectified signal with a peak-to-peak amplitude of 108 V to drive the PZT. Due to the narrow band of PZT resonance frequency, the half-rectified signal was a sinusoidal waveform. Fourier analysis of the acoustic signal produced from the transducer (Figure 1C) showed a central frequency of 380 kHz. Figure 1D shows the acoustic beam profile from a single transducer measured in free water conditions. The beam profile and pressure map were indifferent between the two transducers. Moreover, the acoustic power produced from one transducer was maintained similarly when the second transducer was turned on. The highest pressure was observed around 2 mm from the transducer surface with peak-to-peak pressure of 481 kPa (I_*SPPA*_ = 2.3 W/cm^2^). In the transversal profile, FWHM was 3.2 mm in diameter. A loss of 10% in acoustic pressure was observed due to attenuation caused by the rat skull. The acoustic pressure map after the skull showed the highest peak was maintained around 2 mm from the transducer surface with a minor distortion of the transversal profile (Supplementary Figure 1).

### Low-intensity pulsed ultrasound stimulation modulates brain oscillatory power

**FIGURE 3:**
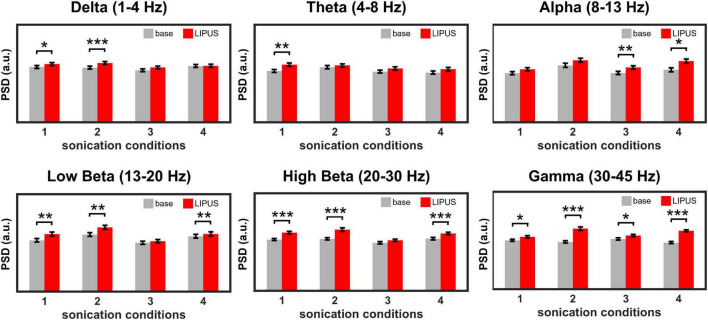
Average power spectral density (PSD) obtained from the right motor cortex during baseline and LIPUS stimulation with unilateral (sonication conditions 1 and 4) and bilateral (sonication conditions 2 and 3) stimulation protocols. (*p < 0.05, **p < 0.01, ***p < 0.001, paired *t*-test.).

Figure 3 shows the effect of LIPUS stimulation on the neural oscillations of the right motor cortex. The overall PSD of the right motor cortex for each sonication condition is shown in Supplementary Figure 2. All four stimulation conditions significantly changed the power of specific brain waves compared to baseline. In detail, a significant increase in power of delta waves was induced by sonication conditions #1 [*t*(1,699) = −2.07, *p* < 0.05] and #2 [*t*(1,699) = −3.31, *p* < 0.001]. Theta wave was significantly elevated by condition #1 [*t*(1,699) = −3.25, *p* < 0.01]. The comparison between pre- and post-stimulation showed that the power of alpha oscillations was significantly increased by sonication conditions #3 [*t*(1,699) = −2.48, *p* < 0.01] and #4 [*t*(1,699) = −2.43, *p* < 0.05]. The power of beta oscillations was significantly increased for sonication conditions #1 [*t*(1,699) = −2.69, *p* < 0.01 for low beta, *t*(1,699) = −4.70, *p* < 0.001 for high beta], #2 [*t*(1,699) = −2.93, *p* < 0.01 for low beta, *t*(1,699) = −4.79, *p* < 0.001 for high beta] and #4 [*t*(1,699) = −3.16, *p* < 0.01 for low beta, *t*(1,699) = −10.23, *p* < 0.001 for high beta]. The power of the gamma brainwaves was also significantly increased in all sonication conditions. [*t*(1,699) = −2.71, *p* < 0.05 for #1; *t*(1,699) = −6.47, *p* < 0.001 for #2; *t*(1,699) = −2.52, *p* < 0.05 for #3; and *t*(1,699) = −15.71, *p* < 0.001 for #4].

### Modulatory effect of bilateral low-intensity pulsed ultrasound stimulation

**FIGURE 4:**
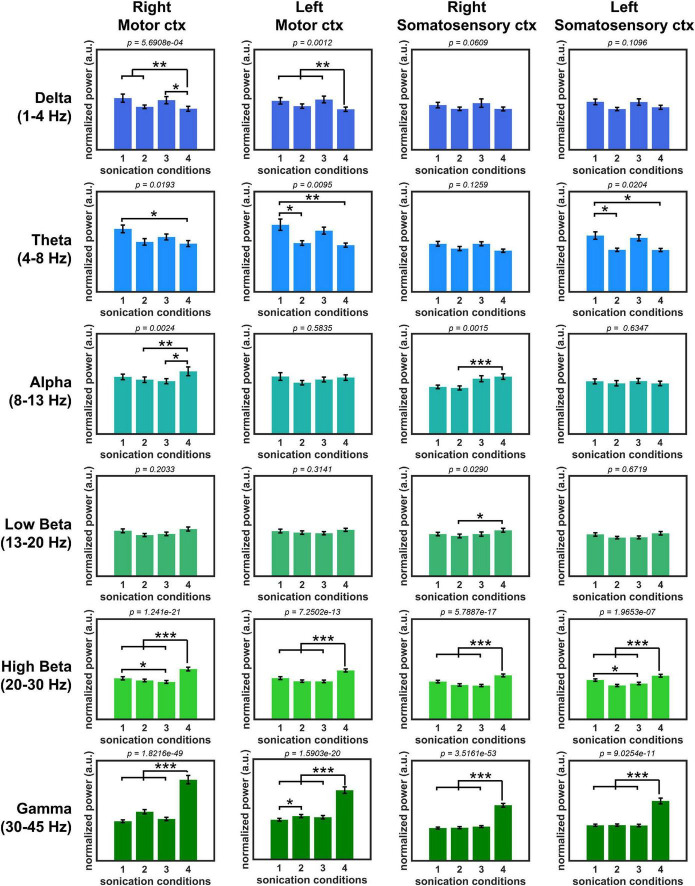
Normalized oscillatory power with four sonication conditions. (*p < 0.05, **p < 0.01, ***p < 0.001, Kruskal–Wallis, non-parametric one-way ANOVA followed by the Tukey–Kramer *post hoc* analysis.).

The normalized PSD of all sonication conditions is shown in Figure 4. PSD comparison showed a statistically significant difference between unilateral sonication conditions #1 (1 kHz PRF and 50% DC) and #4 (100 Hz PRF and 5% DC) in the power of delta (motor cortex from both hemispheres showed a higher power during #1, both *p* < 0.01), theta (motor cortex from both hemispheres, and left somatosensory cortex showed a higher power during #1, both *p* < 0.05), high beta (motor and somatosensory cortices from both hemispheres showed a lower power during #1, both *p* < 0.01), and gamma oscillations (motor and somatosensory cortices from both hemispheres showed a lower power during #1, both *p* < 0.01). We also compared the PSD changes induced by unilateral and bilateral stimulation to observe the modulatory effect associated with the functional interhemispheric connection. The effect of unilateral stimulation of the right primary motor cortex with sonication condition #1 (1 kHz PRF and 50% DC) on PSD was significantly changed by the simultaneous stimulation of the contralateral primary motor cortex with 100 Hz PRF and 5% DC (sonication condition #2). The power of theta oscillations (left motor cortex) significantly (*p* < 0.05) decreased, while gamma power significantly increased (*p* < 0.05) during sonication #2. The PDS of high beta power recorded from the right motor and left somatosensory cortices significantly (*p* < 0.05) decreased when 1 kHz PRF and 50% DC sonication were applied bilaterally (sonication condition #3) compared to unilateral 1 kHz PRF and 50% DC sonication (sonication condition #1). The effect of unilateral 100 Hz PRF and 5% DC was also changed by simultaneous sonication of the contralateral motor cortex with a 1 kHz PRF and 50% DC pulsing scheme. A significant (*p* < 0.05) increase in delta (left motor cortex) and a decrease in alpha, high beta, and gamma power were observed during bilateral stimulation #2 compared to unilateral stimulation #4.

## Discussion

In this work, we developed a wireless LIPUS system for bilateral brain stimulation, characterized the system capability, and validated the acoustic stimulation *in vivo* over the motor cortex on the anesthetized rats. The system characterization showed that the developed device offered a level of acoustic energy that matched previous neuromodulation studies (Puts et al., 2015; Niu et al., 2022). The designed device also showed the capability to change sonication parameters (PRF, DC, ISI, and SD) remotely, offering a rapid adjustment of the stimulation parameters according to the application. Powered by a battery, the developed system possesses portable for rat size and weight. Considering stimulation protocols used in the present study, a battery with 900 mAh provides 40 min of unilateral or 25 min of bilateral sonication with pulsing. The battery with higher capacitance can be used for a longer SD, but the system’s total weight will be increased. It is important to mention that due to the internal resistance of PZT, prolonged continuous sonication may cause the thermal elevation of the ultrasound transducer, leading to thermal injury to tissues or permanent damage to the transducer. Thus, we do not recommend using a DC over 50% or a pulse duration longer than 10 ms. Sub-megahertz ultrasound frequency selected in this study offered excellent transmission through the rat skull. Based on the acoustic map and location of transducers, along with acoustic simulation from our previous work (Kim et al., 2020), the developed system produced an ultrasound field presumably concentrated within the motor cortex.

*In vivo* evaluation of the developed LIPUS system was performed based on power change of brain oscillations. Despite the potential of the LIPUS device to be used on awake rats, the anesthetized animal model was chosen to avoid any confounding brain activities or motion artifacts during the awake state. Four sonication scheme was examined in this study. Previous studies showed that the specific LIPUS parameters produced a unique stimulation effect (Zhang et al., 2021). Short DC (<10%) with slow PRF (<100 Hz) tended to lead to the inhibitory effect, while DC above 30% with PRF around 1 kHz is commonly used for excitatory application (Yoon et al., 2019). Recent work with single neuron recording and optogenetic intervention also demonstrated that the excitatory neurons are more sensitive to higher PRF protocols, while the response from inhibitory neurons is indifferent regardless of PRF (Yu et al., 2021). Therefore, two distinct stimulation parameters (1 kHz PRF and 50% DC, presumably excitatory, and 100 Hz PRF and 5% DC, presumably inhibitory) were chosen in this study. Here, the power density analyses of EEG signal showed that the 1 kHz PRF and 50% DC stimulation produced a higher power in slow brain oscillations (delta and theta) and lower power in high beta and gamma oscillations compared to 100 Hz PRF and 5% DC stimulation. Although spectral power change does not certainly prove which sonication parameters suppress or excite, a decrease in beta power was associated with motor execution (Brinkman et al., 2014). Thus, lower power in beta oscillation may provide additional support that 1 kHz PRF and 50% DC sonication had an excitatory property.

Our observation showed that bilateral sonication produced a significantly different effect on PSD compared to unilateral sonication. The effect of unilateral stimulation of the right primary motor cortex with presumably excitatory (sonication condition 1) was significantly altered by contralateral presumably inhibitory sonication in PSD theta and gamma. When presumably excitatory sonication was applied to both hemispheres, a power of high beta oscillation was only changed compared to unilateral “excitatory” sonication. The broader range of PSD was substantially affected by contralateral sonication during presumably inhibitory sonication. The comparison of unilateral “inhibitory” with bilateral stimulation showed a significant difference in the power of delta, alpha, high beta, and gamma brainwaves. Meanwhile, the modulatory effect of bilateral conditions #2 and #3 showed no difference in oscillatory bands between each other, even though the total acoustic energy deposition of stimulation #3 (1 kHz PRF and 50% DC on each hemisphere) is higher compared to bilateral stimulation #2 (100 Hz PRF and 5% DC). Thus, the above observation may imply that changes in PSD are not a simple outcome of differences in acoustic energy deposition within the brain but rather a unique feature of stimulation schemes. It is also important to mention that considering the diameter of transducers (7 mm) and the geometry of the rat’s skull, the intersection of two acoustic beams within the brain would be negligible in the present study. Meanwhile, if necessary, the developed system can be adapted for the dual-crossed transducer technique to achieve high spatial precision by replacing the current ultrasound transducers with the ones with a longer focal distance (Kim et al., 2021).

Many previous studies of bilateral brain stimulation concentrated on implementing identical stimulation protocols with either the excitatory or inhibitory effect (Rogasch et al., 2014). However, our results imply that some possibility of using the same stimulation protocol on the bilateral brain region may not effectively induce additional effects on the brain in some cases compared to unilateral brain stimulation. In contrast, the bilateral LIPUS stimulation with two different stimulation protocols on each hemisphere showed a modulatory effect on both slow theta and fast gamma brain oscillations compared to the unilateral stimulation.

A statistically significant difference between EEG signals obtained after uni- and bilateral LIPUS indicates the modulatory effect within functional interhemispheric communication. Functional interhemispheric communication is primarily carried out through the corpus callosum. Substantial evidence demonstrated that the stimulation of a brain region in one hemisphere affects the neural activity of the contralateral brain area (Bloom and Hynd, 2005). Interhemispheric inhibition in the motor cortex was observed in healthy subjects as a process in which an increase in activity of one motor cortex inhibited a motor evoked potential initiated by the opposite motor cortex (Ferbert et al., 1992). In unilateral stroke, inhibition between the cerebral hemispheres is essential in post-stroke rehabilitation. The impaired interhemispheric connection after stroke reduces inhibition from the lesioned area onto the contralateral brain region. The overexcitation of the contralesional hemisphere produces aberrant interhemispheric inhibition hindering poststroke recovery (Gazzaniga, 2000). On the other side, the external inhibition of the contralesional hemisphere using TMS was already implemented as a treatment procedure for stroke patients (Kirton et al., 2010; Boddington and Reynolds, 2017). In previous publications, the unilateral LIPUS-mediated excitation of stroke-affected regions also showed a neuroprotective effect (Liu et al., 2019; Baek et al., 2020). However, most stroke studies with TMS or LIPUS were performed with unilateral stimulation of ipsilesional or contralesional hemispheres or bilateral stimulation with identical protocol on both hemispheres. Incorporating bilateral LIPUS stimulation for simultaneous suppression and/or excitation of hemispheres, based on the structural reserve after stroke, could provide a superior therapeutic benefit. The proposed bilateral LIPUS system could be utilized in future animal studies to determine the optimal stimulation protocol for stroke-affected brains.

## Data availability statement

The dataset used and analyzed during this study are available from the corresponding author upon request.

## Ethics statement

The animal study was reviewed and approved by the Institutional Animal Care and Use Committee (IACUC) of the Korea Institute of Science and Technology.

## Author contributions

All authors made substantial contributions to the study concept, design, and/or the acquisition of data, analysis, and/or data interpretation. EK was in charge of hardware development. JK and SL performed the animal experiments and data analysis. EK and JK prepared the manuscript. HK led the study. All authors have read and approved the final manuscript.
