# Supplementary material for: Development of a wireless ultrasonic brain stimulation system for concurrent bilateral neuromodulation in freely moving rodents
Source: Front Neurosci. 2022 Sep 23;16:1011699. doi: 10.3389/fnins.2022.1011699 (PMC9539445; doi:10.3389/fnins.2022.1011699)
Supplement: Supplementary file 1 [file Image_1.pdf]

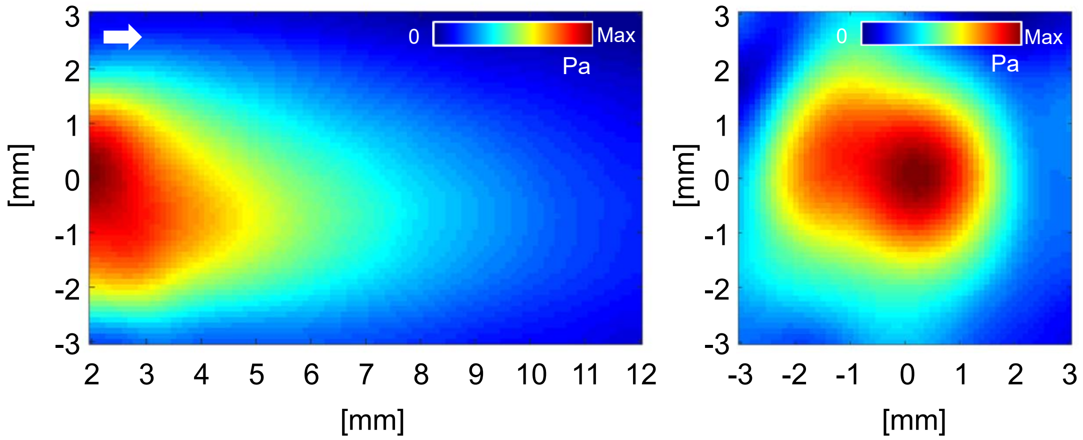

Figure S1. The acoustic beam profile of the in-house built transducer with a rat skull in front of the exit plane. The longitudinal pressure map is on the left, and the transverse profile is on the right (measured 2 mm away from the transducer surface). The white arrow shows the direction of sonication.

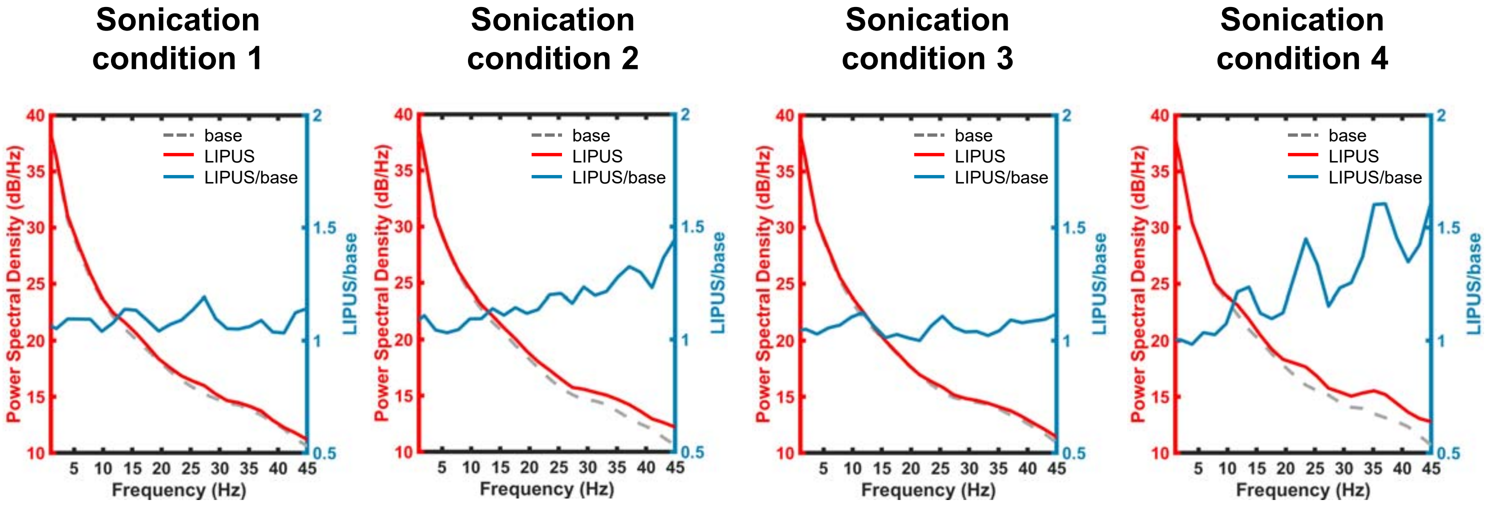

Figure S2. The overall power spectral density (PSD) of the right motor cortex for each sonication condition.
